# Supplementary material for: Preclinical assessment of splicing modulation therapy for ABCA4 variant c.768G>T in Stargardt disease
Source: Commun Med (Lond). 2025 Jan 21;5:25. doi: 10.1038/s43856-024-00712-7 (PMC11751084; doi:10.1038/s43856-024-00712-7)
Supplement: Supplementary file 2 — Supplementary Information [file 43856_2024_712_MOESM2_ESM.docx]

**Preclinical assessment of splicing modulation therapy for *ABCA4* variant c.768G>T in Stargardt disease**

Running title: Splicing modulation of *ABCA4* c.768G>T

Dyah W. Karjosukarso (1), Femke Bukkems (1,2), Lonneke Duijkers (1), Tomasz Z. Tomkiewicz (1), Julia Kiefmann (1), Andrei Sarlea (3), Sander Bervoets (4), Irene Vázquez-Domínguez (1), Laurie L. Molday (5), Robert S. Molday (5), Mihai G. Netea (3), Carel B. Hoyng (2,6), Alejandro Garanto (1,7), Rob W.J. Collin* (1,2)

1. Department of Human Genetics, Radboud University Medical Center, Nijmegen, the Netherlands.
2. Astherna B.V., Nijmegen, The Netherlands.
3. Department of Internal Medicine and Radboud Center for Infectious Diseases, Radboud University Medical Centre, Nijmegen, the Netherlands
4. Radboudumc Technology Center Bioinformatics, Radboud University Medical Center, Nijmegen, the Netherlands
5. Department of Biochemistry and Molecular Biology, The University of British Columbia, Vancouver, Canada
6. Department of Ophthalmology, Radboud University Medical Center, Nijmegen, the Netherlands
7. Department of Pediatrics, Amalia Children´s Hospital, Radboud University Medical Center, Nijmegen, the Netherlands


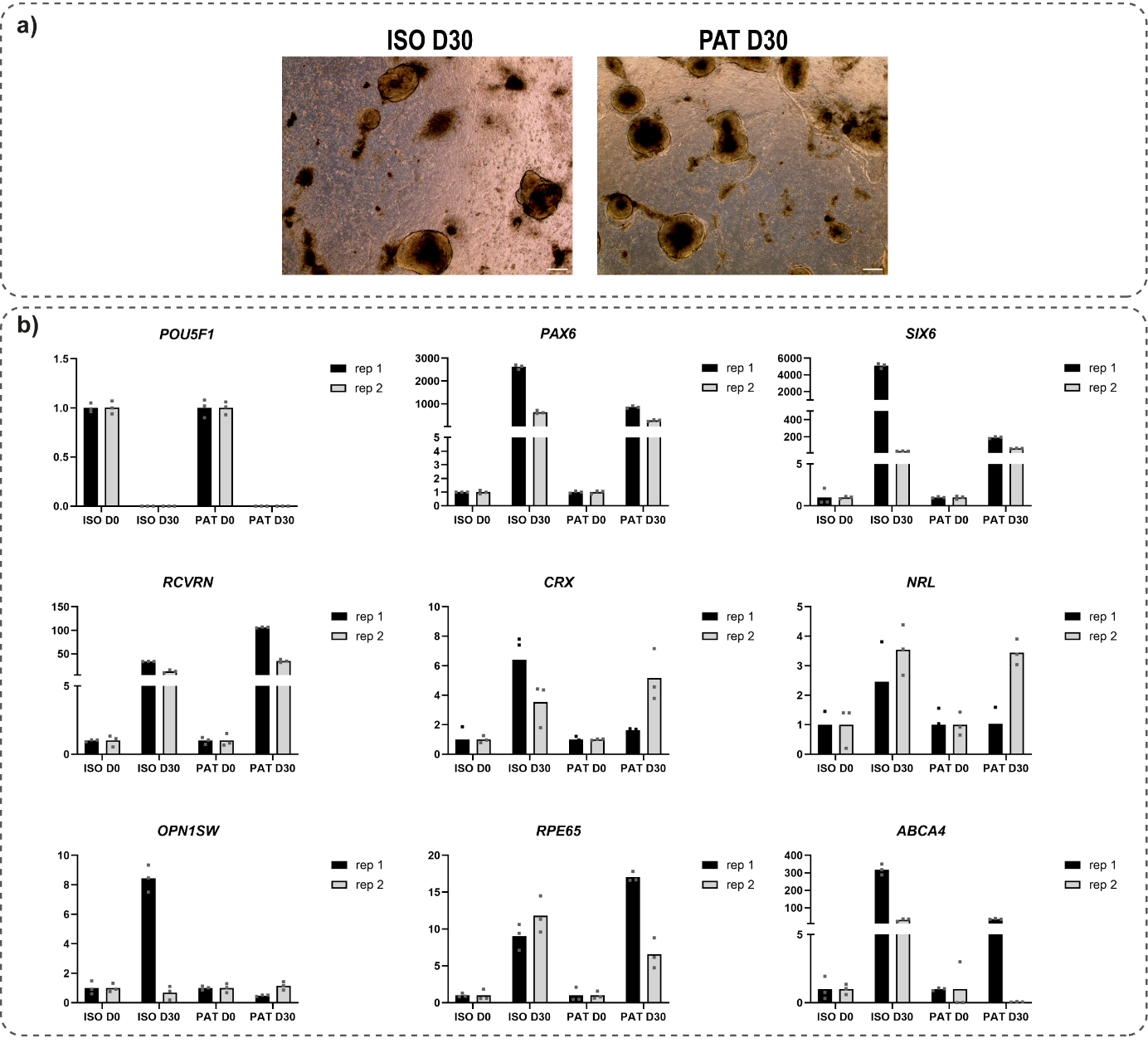


*Supplementary Figure 1 Characterization of PPCs.*

a) Representative phase contrast images of PPCs at the end of the differentiation (day 30). Scale bar equals to 50 μm. b) Expression level of the pluripotency marker (*POU5F1*), photoreceptor markers (*PAX6*, *SIX6*, *RCVRN*, *CRX*, *NRL*, *OPN1SW*), retinal pigmented epithelium marker (*RPE65*) and gene of interest (*ABCA4*) measured by qPCR. The expression level is illustrated as fold-change relative to day 0 of the differentiation (iPSC state). The two independent differentiations are depicted separately as rep 1 and rep 2. Each dot represents the technical replicates of the measurement.


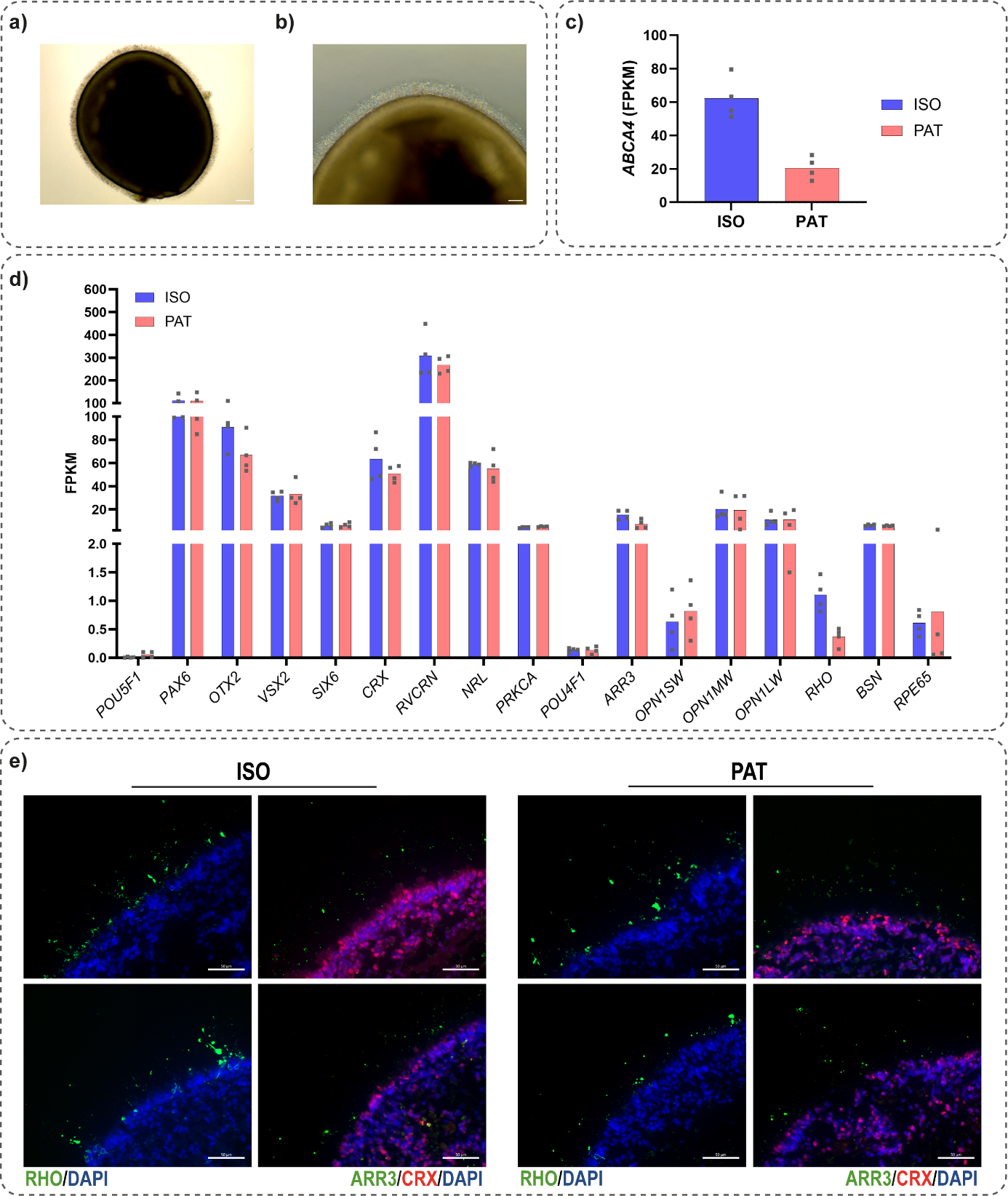


*Supplementary Figure 2 Characterization of ROs.*

a) Representative phase contrast images of mature ROs (~day 230). Scale bar equals 100 μm. b) Higher magnification of a), scale bar equals 200 μm. c) FPKM-normalized expression of *ABCA4* in isogenic control vs patient-derived ROs (~day 240). d) FPKM-normalized expression of pluripotency marker (*POU5F1*), various neuroretina and photoreceptor markers as well as retinal pigment epithelium marker (*RPE65*) in isogenic control and patient derived ROs (~day 240). FPKM stands for fragments per kilobase of exon per million mapped fragments. c) and d) Each dot represents one biological replicate shown in Figure 5. e) Immunohistochemistry analysis of photoreceptor markers in isogenic control and patient derived ROs (day ~220-240). Representative images from two biological replicates are shown. Scale bar equals to 50 μm.


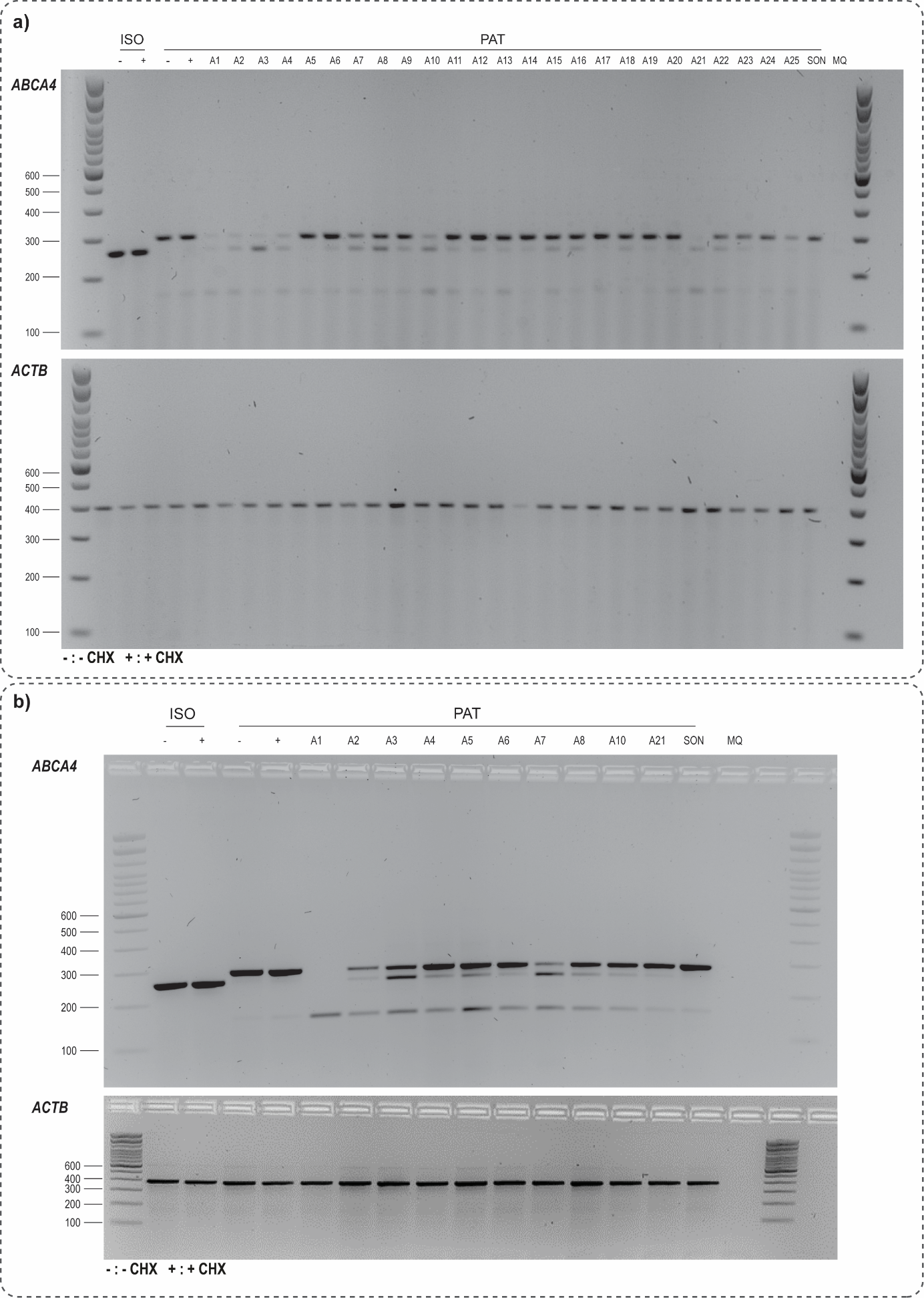


*Supplementary Figure 3 AON screening in PPCs and ROs.*

a) Full-image of representative RT-PCR analysis shown in Figure 2b. b) Full-image of representative RT-PCR shown in Figure 2d.


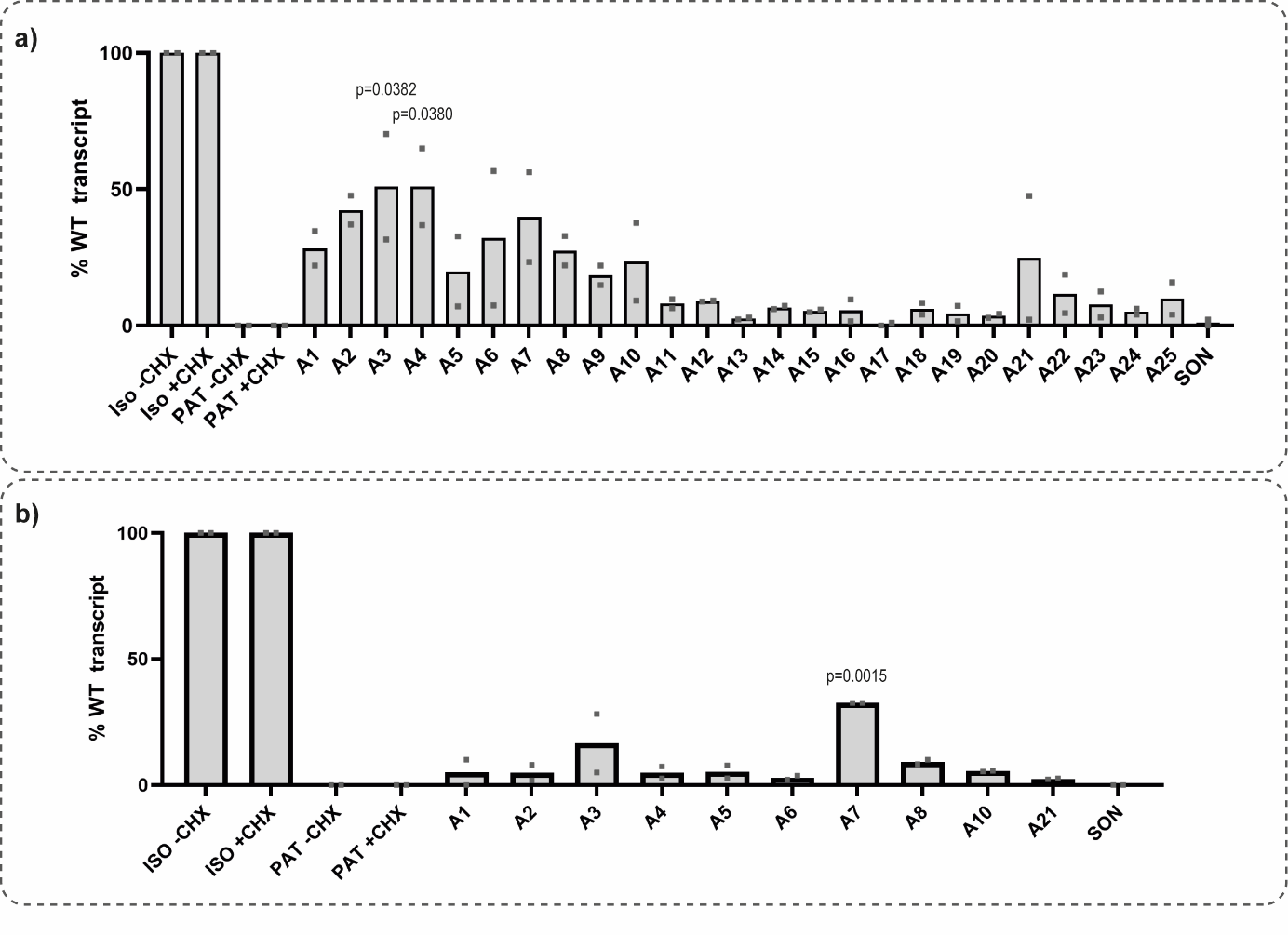


*Supplementary Figure 4 AON screening in PPCs and ROs.*

1. Agilent Tapestation quantification of WT transcript level in PPCs treated with 2’MOE/PS AONs targeting c.768G>T for 10 d at 5 μM final concentration (RT-PCR Figure 2b). b) Agilent Tapestation quantification of WT transcript level in ROs treated with 2’MOE/PS AONs targeting c.768G>T for 10 d at 10 μM final concentration (RT-PCR Figure 2d). a) and b) The %WT transcript level illustrated is based on the ratio of aberrant, correct, and exon 6 deletion transcripts in each sample, with total signal quantified as 100%. The quantification was performed on one PCR product per biological replicate (n=2). Significance was calculated by one-way ANOVA followed by post-hoc Dunnett test comparing each AON-treated sample to SON. Only AON and SON-treated samples are included in the statistical analysis.


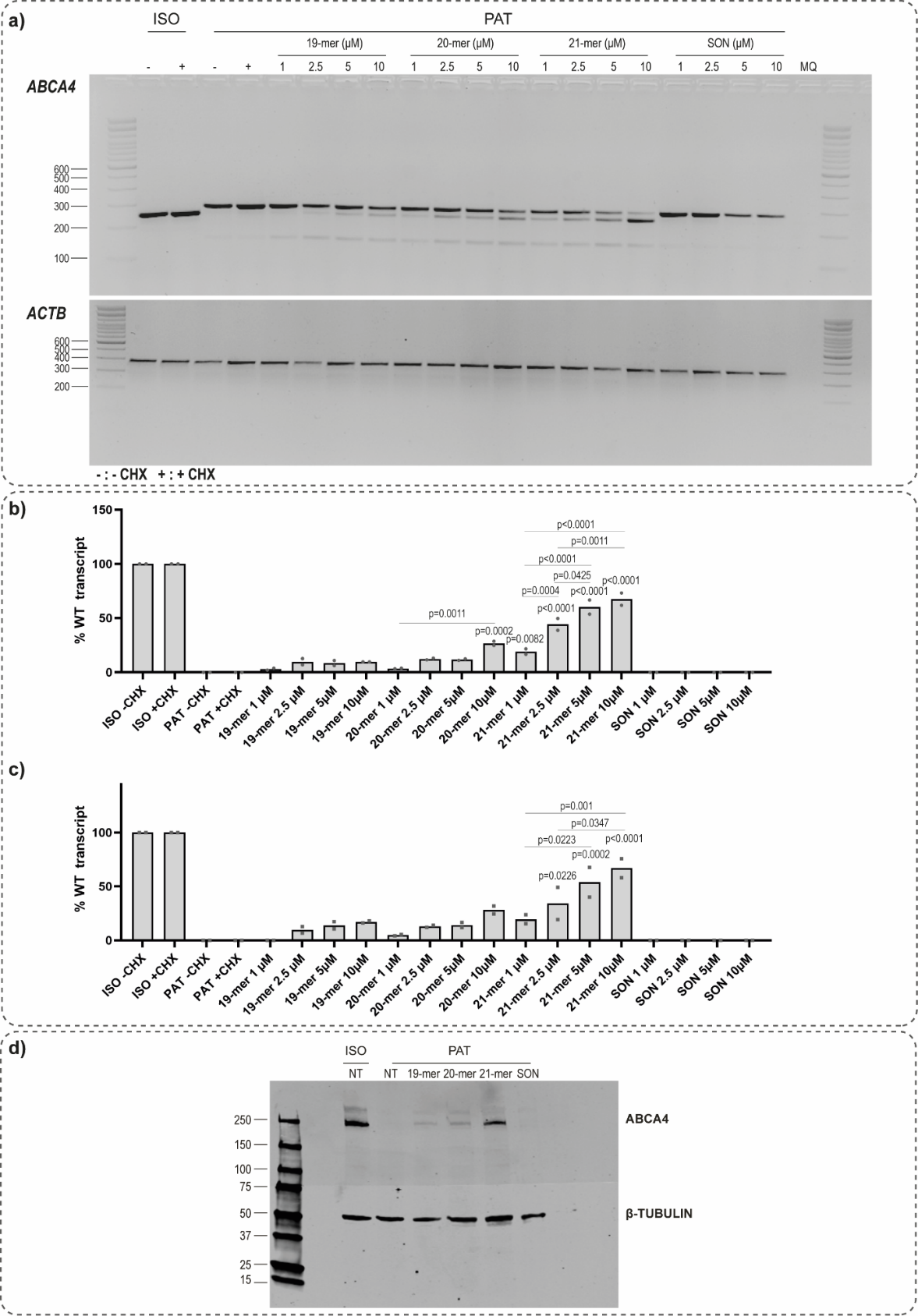


*Supplementary Figure 5 A7 21-mer performed better than the shorter versions in a dose-dependent manner.*

a) Full image of representative RT-PCR analysis shown in Figure 3a. b) Fiji semi-quantification and c) Agilent Tapestation quantification of WT transcript level based on RT-PCR analysis in Figure 3a. b) and c) The %WT transcript level illustrated is based on the ratio of aberrant, correct, and partial exon 6 deletion transcripts in each sample, with total signal quantified as 100%. The quantification was performed on one PCR product per biological replicate (n=2).Significance was calculated by one-way ANOVA followed by post-hoc Dunnett test comparing each AON-treated sample to SON. d) Full image of representative Western blot analysis shown in Figure 3b.


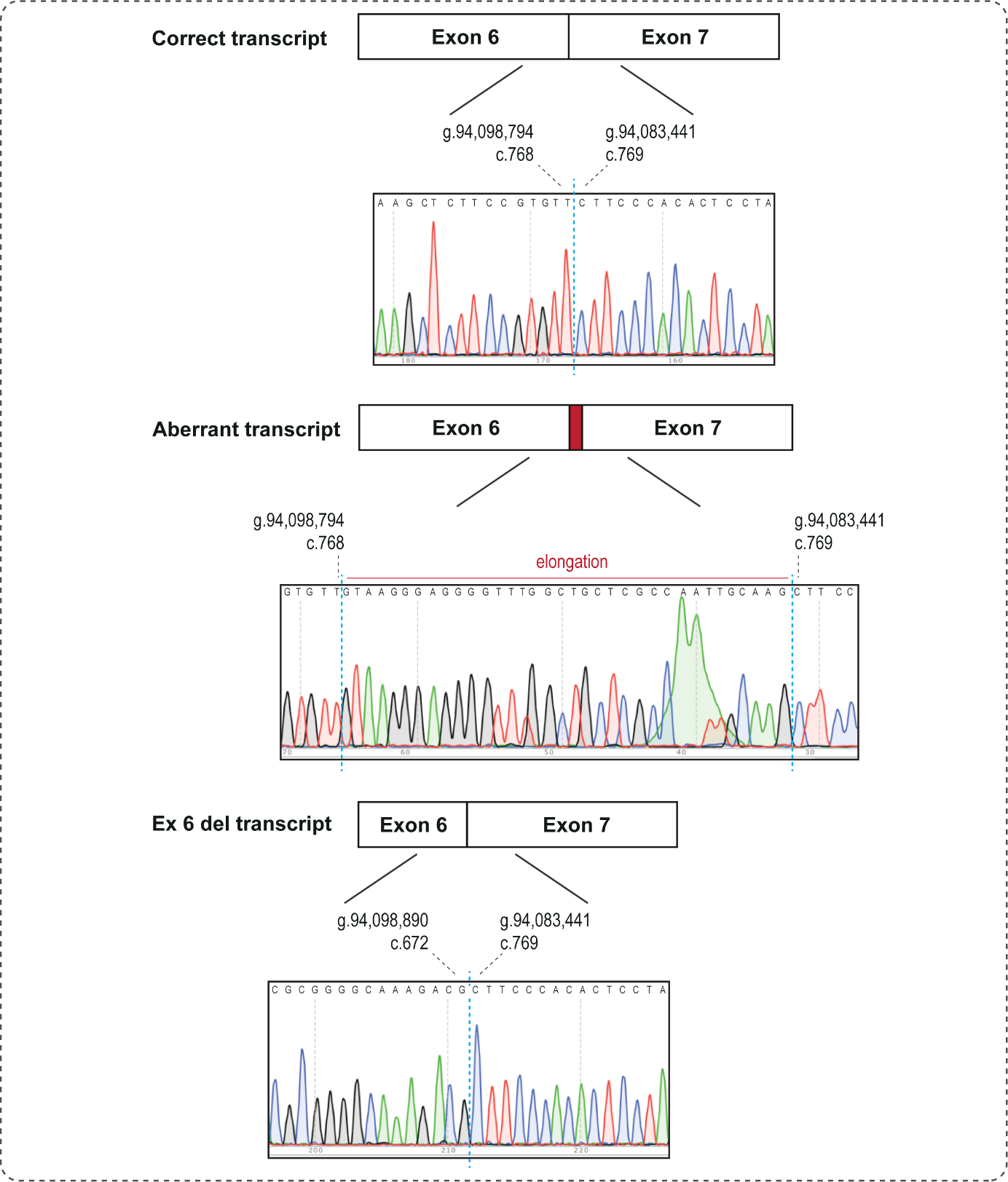


*Supplementary Figure 6 Representative Sanger sequences of the detected transcripts in RT-PCR analysis.*

These transcripts correspond to those detected in Figure 2, Figure 3 and Figure S7.


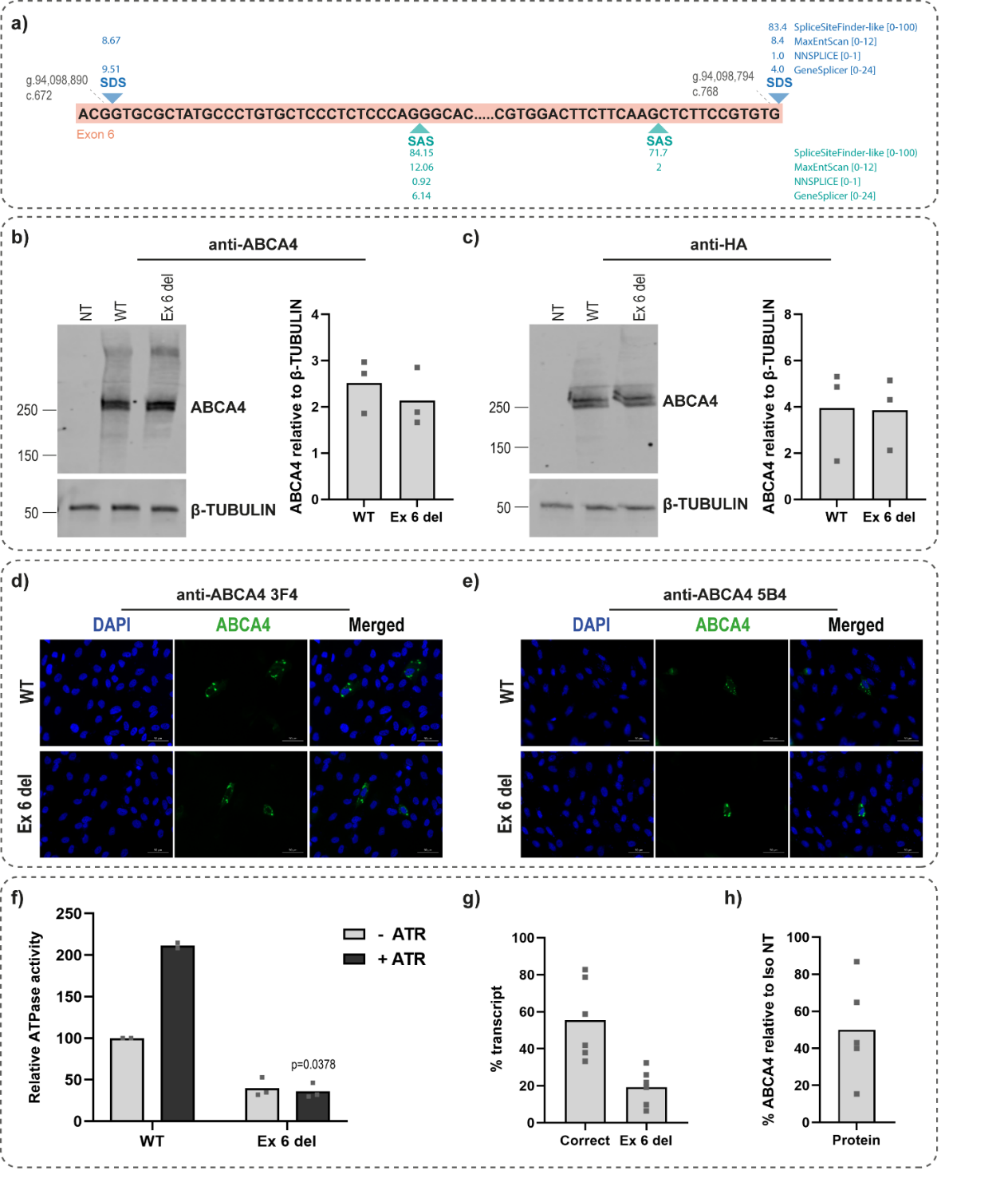


*Supplementary Figure 7 The effect of partial deletion of ABCA4 exon 6.*

a) A schematic overview of the splice donor site (SDS) and splice acceptor site (SAS) from the deletion point to the end of *ABCA4* exon 6. b) and c) Western blot analysis of HEK293T cells transfected with constructs containing wild-type full-length (WT) and partial exon 6 deletion (Ex 6 del) *ABCA4* using antibody against HA-tag (b) and ABCA4 (c) included in the construct. Error bars indicate standard deviation from three biological replicates. d) and e) Localization study in hTERT RPE-1 cells transfected with constructs containing wild-type full-length (WT) and partial exon 6 deletion (Ex 6 del) *ABCA4* using different monoclonal antibodies targeting ABCA4 (3F4 in e) and 5B4 in e)). f) Relative ATPase activity measured in HEK293T cells transfected with constructs containing wild-type full-length (WT) and partial exon 6 deletion (Ex 6 del) *ABCA4*, with and without all-trans-retinal (ATR) stimulation. Each dot indicates one biological replicate. g) A summary % correct transcript and ex 6 del transcript in all ROs treated with A7 21-mer 10 µM A7 21-mer for 10 days (i.e. Figure 2, Figure 3 and Figure S7). h) A summary % ABCA4 relative to WT in all ROs treated with 10 µM A7 21-mer for 30 days (i.e. Figure 3 and Figure S7). g) and h) Each dot represents 1 biological replicate.


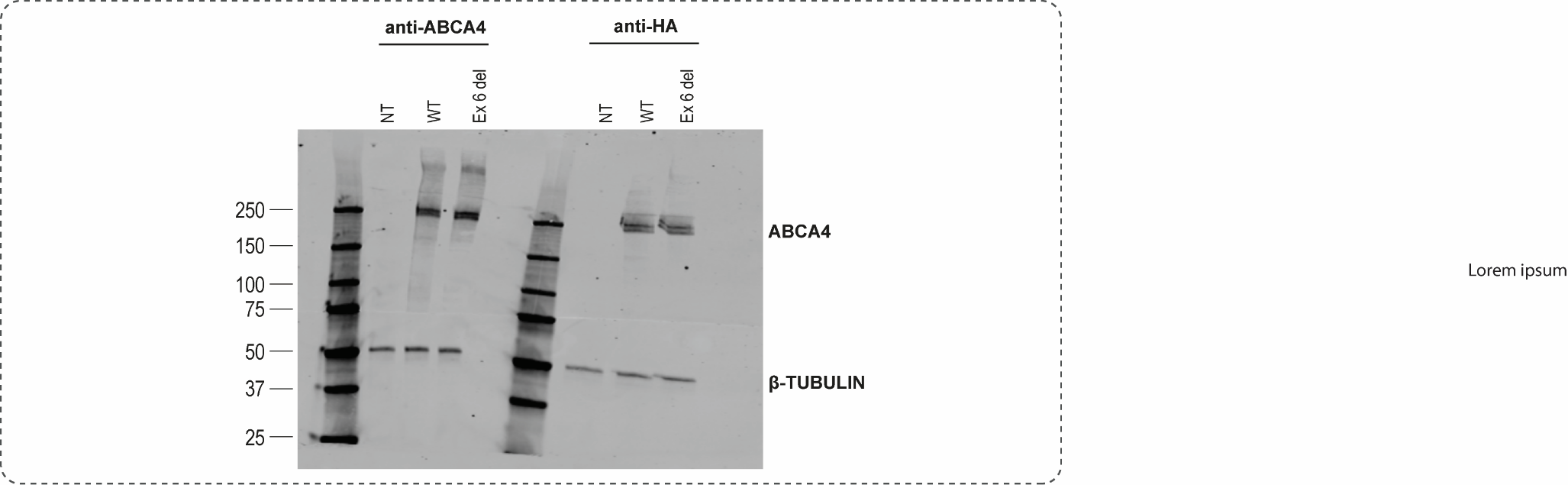


*Supplementary Figure 8 The effect of partial deletion of ABCA4 exon 6.*

Full image of representative Western blot analysis shown in Supplementary Figure 7b and c.


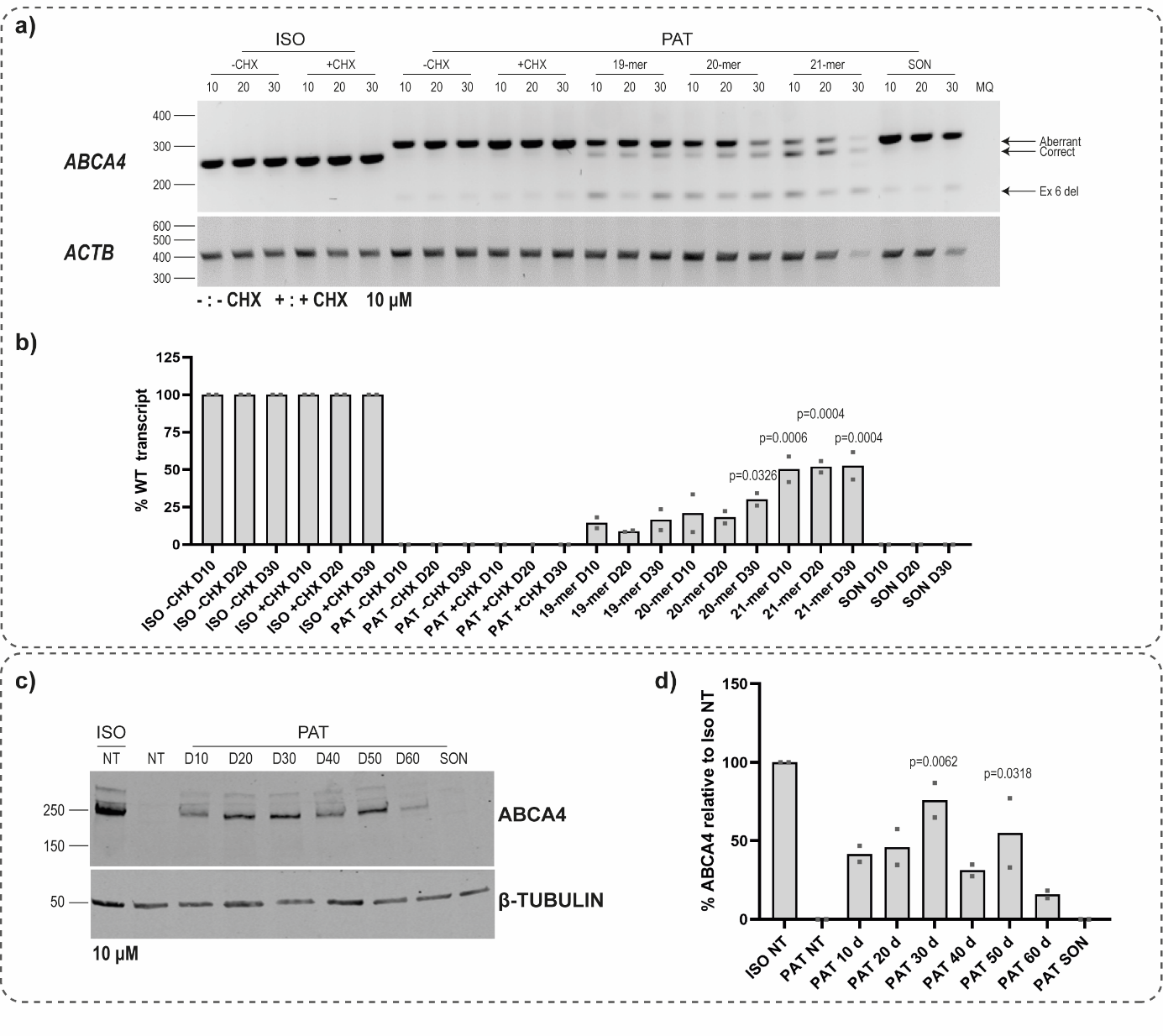


*Supplementary Figure 9 Treatment duration influenced rescue at protein level, but not at transcript level in ROs.*

a) RT-PCR analysis of ROs treated with different lengths of A7 for 10, 20 and 30 days. ISO denotes isogenic control and PAT represents patient-derived ROs. Three different transcripts were detected, aberrant transcript containing 35-nt exon 6 elongation, correct/wild-type transcript, and ex6 del referring to a transcript containing 96 bp deletion of exon 6 (c.673-c.768). *ACTB* was used as loading control. b) Fiji semi-quantification quantification of WT transcript level based on RT-PCR analysis in a). The %WT transcript level illustrated is based on the ratio of aberrant, correct, and partial exon 6 deletion transcripts in each sample, with total signal quantified as 100%. Each dot denotes the mean of technical replicates of one biological replicate. Significance was calculated by one-way ANOVA followed by post-hoc Dunnett test comparing each AON-treated sample to SON. c) Western blot analysis of ROs treated with A7 21-mer for different duration at 10 μM final concentration. d) Fiji semi-quantification of ABCA4 expression based on the band at ~250 kDa, normalized to β-TUBULIN expression, relative to Isogenic control. Each dot denotes the mean of technical replicates of one biological replicate. Significance was calculated by one-way ANOVA followed by post-hoc Dunnett test comparing each AON-treated sample to SON.


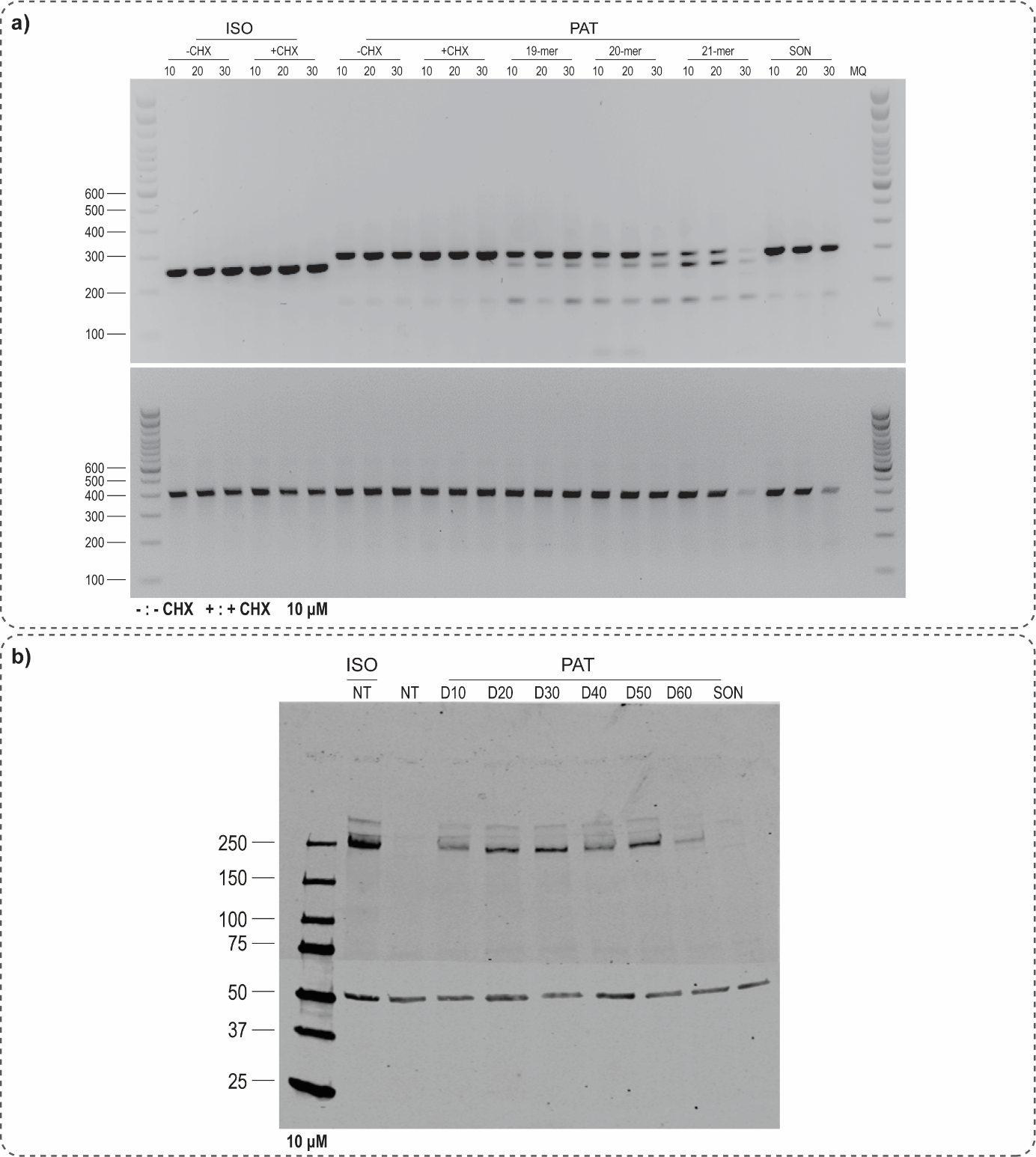


*Supplementary Figure 10 Treatment duration influenced rescue at protein level, but not at transcript level in ROs.*

a) Full image of representative RT-PCR analysis shown in Supplementary Figure 9a. b) Full image of representative Western blot analysis shown in Supplementary Figure 9c.


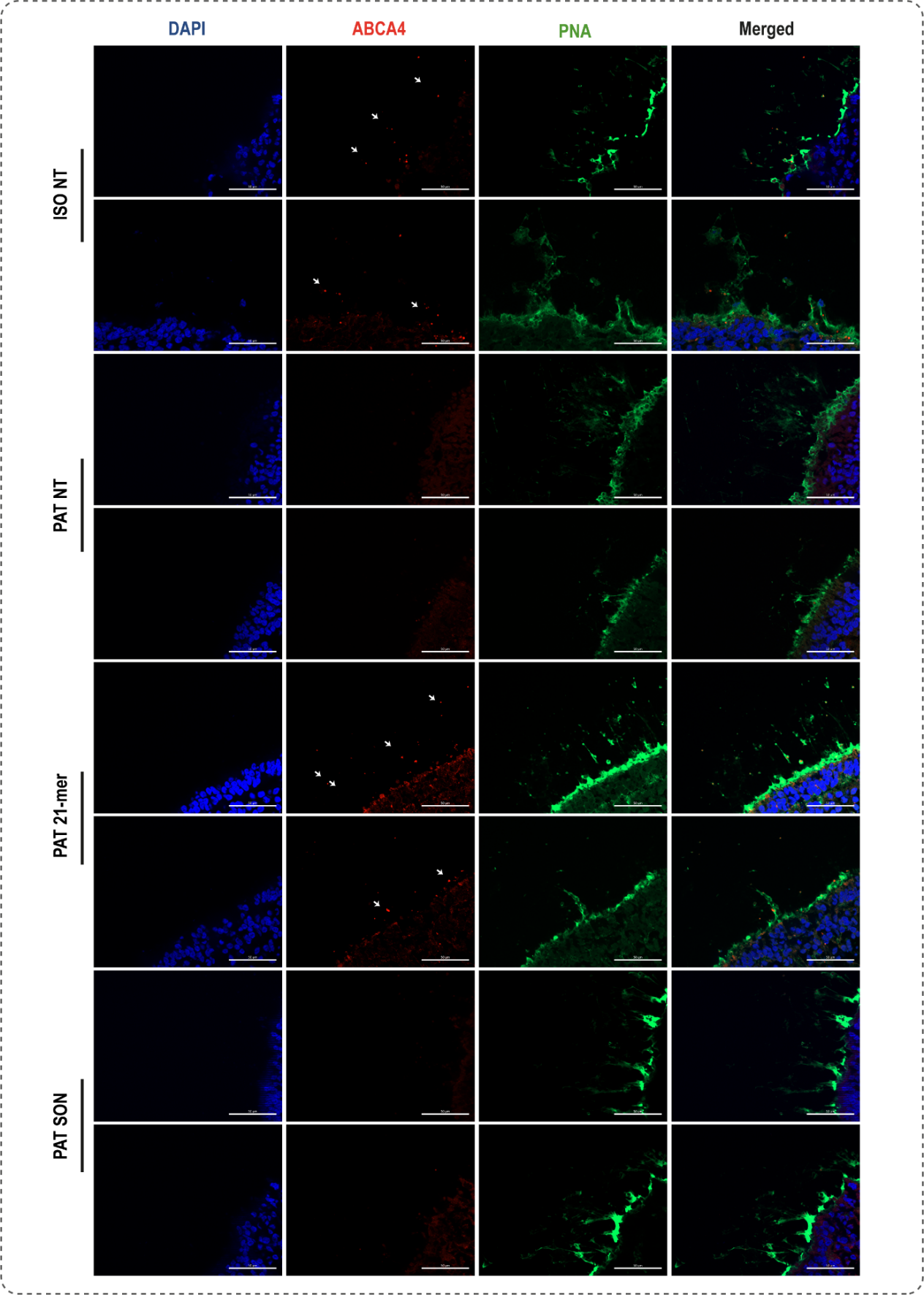


*Supplementary Figure 11 Rescued ABCA4 is expressed in the outer segments of ROs upon 30 d treatment with 10 μM A7 21-mer.*

Immunohistochemistry analysis of ABCA4 localization (red, 5B4 antibody), PNA (green) was used to mark the outer segments of the ROs and DAPI (blue) was used as nuclear marker. Representative images from three biological replicates are shown. Scale bar equals to 50 μm.


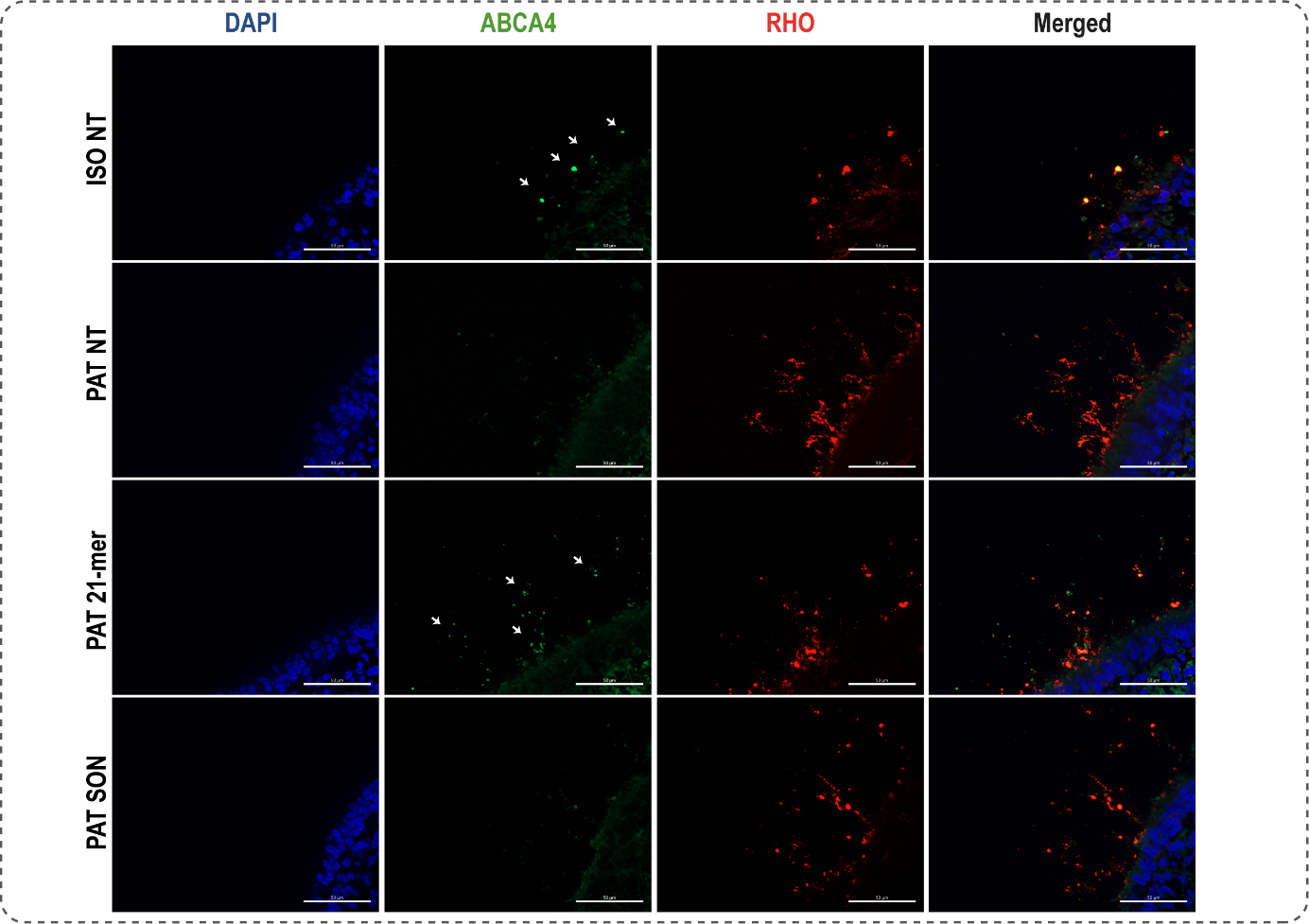


*Supplementary Figure 12 Rescued ABCA4 is expressed in the outer segments of ROs upon 30 d treatment with 10 μM A7 21-mer.*

Immunohistochemistry analysis of ABCA4 localization (green, 3F4 antibody), RHO (red) was used to mark the outer segments of the ROs and DAPI (blue) was used as nuclear marker. Representative images from three biological replicates are shown. Scale bar equals to 50 μm.


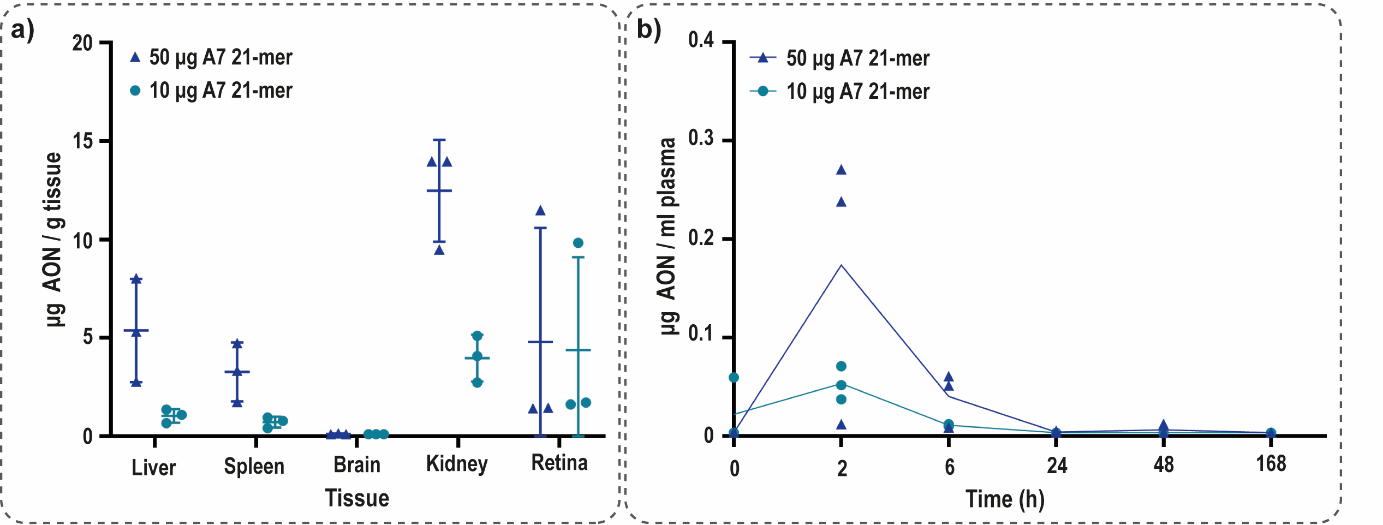


*Supplementary Figure 13 Systemic exposure of A7 21-mer following intravitreal injection.* a) Quantification of A7 21-mer in other organs 1 week (168 h) post-injection by probe-based ELISA. b) Quantification of A7 21-mer in plasma at different time-points by probe-based ELISA. Each dot represents measurement from one mouse.


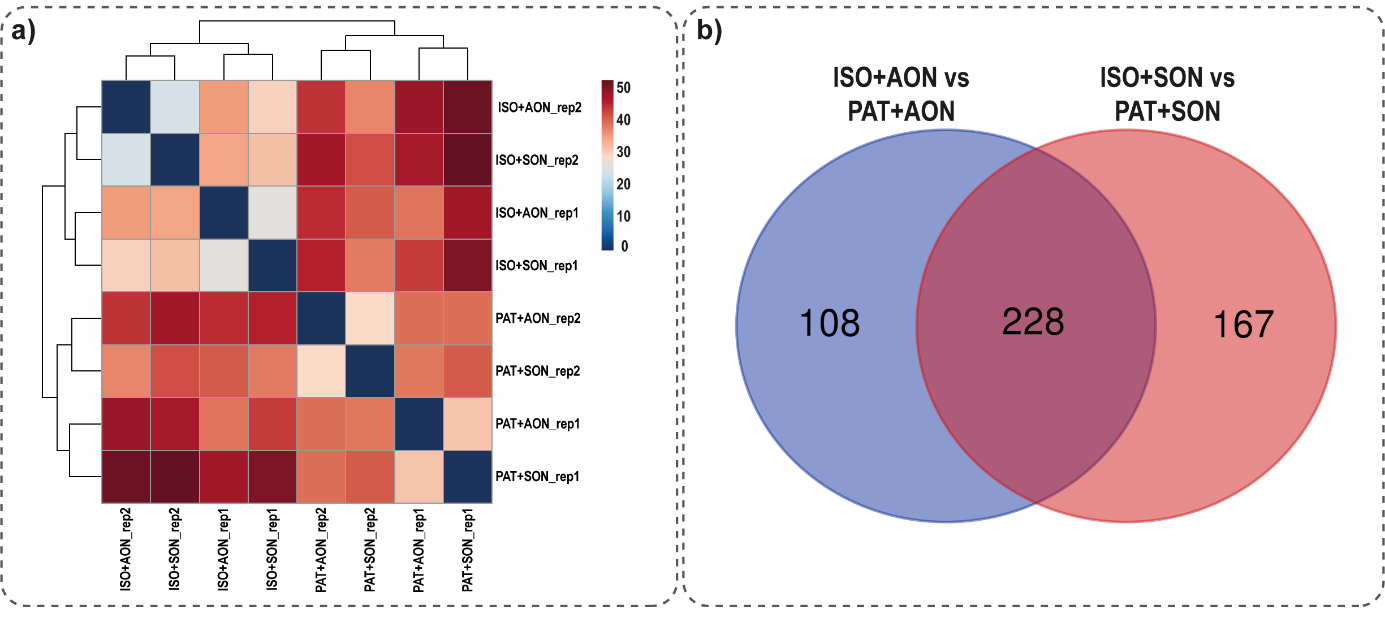


*Supplementary Figure 14 RNA-seq analysis of isogenic and patient-derived ROs following A7 21-mer and SON treatment.*

a) Heatmap of Euclidean distance between the different experimental conditions. b) Venn diagram depicting the overlapping differentially expressed genes observed in pairwise comparison ISO+AON *vs* PAT+AON and pairwise comparison ISO+SON *vs* PAT+SON. A)-B) ROs were treated for 10 d prior to analysis. ISO: isogenic control, PAT: patient-derived ROs, AON: A7 21-mer, SON: sense oligonucleotides of A7 21-mer.


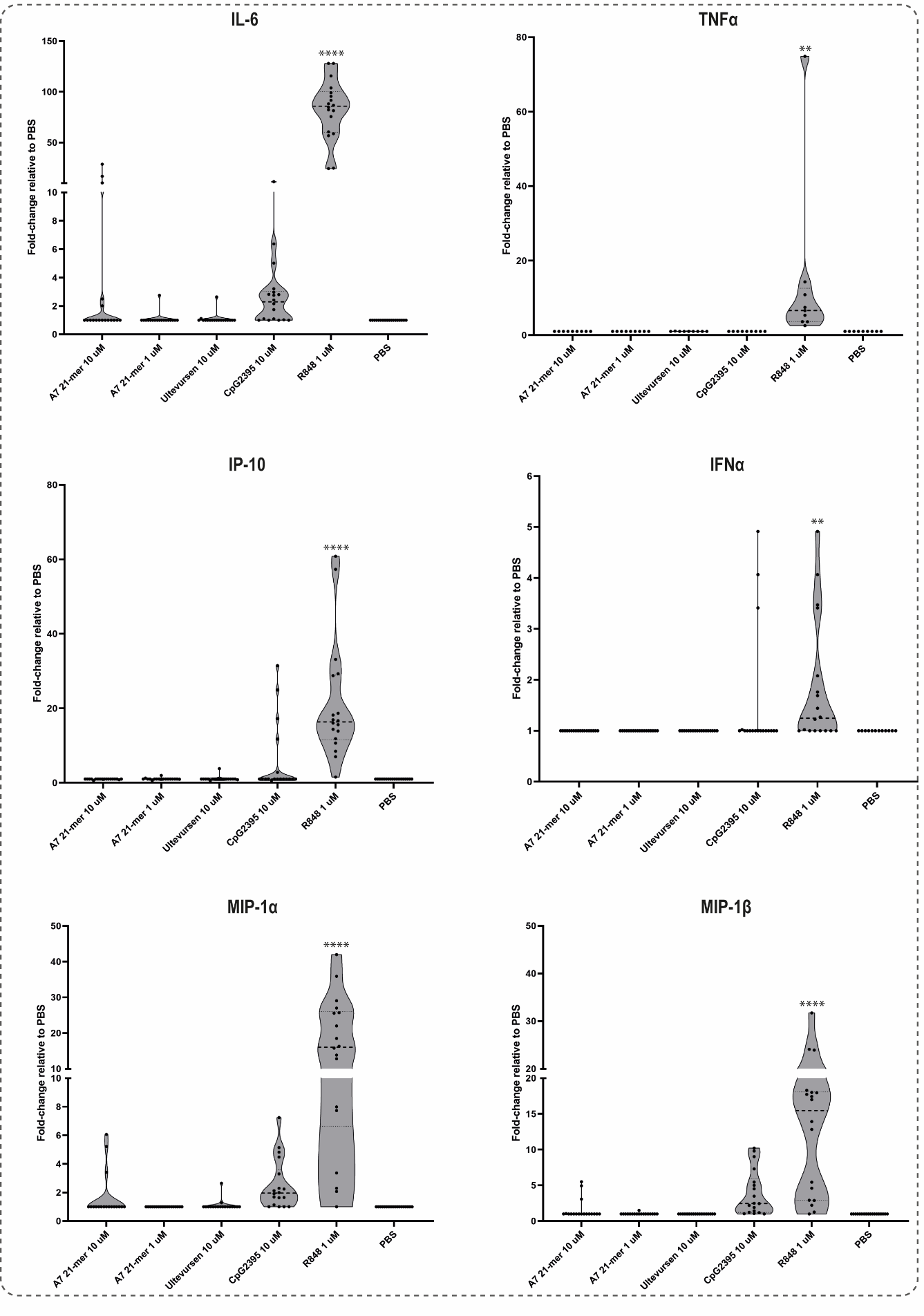


*Supplementary Figure 15 Cytokines and chemokines release upon 24 h PBMC stimulation with A7 21-mer.*

Data is presented as fold-change relative to vehicle (PBS). Each dot represent one healthy donor included in the study. Significance was calculated by one-way ANOVA followed by post-hoc Dunnett test comparing each AON-treated sample to vehicle (PBS). The sign * denotes p-value <0.05 , ** p-value <0.01, *** p-value <0.001 and **** p-value <0.0001.


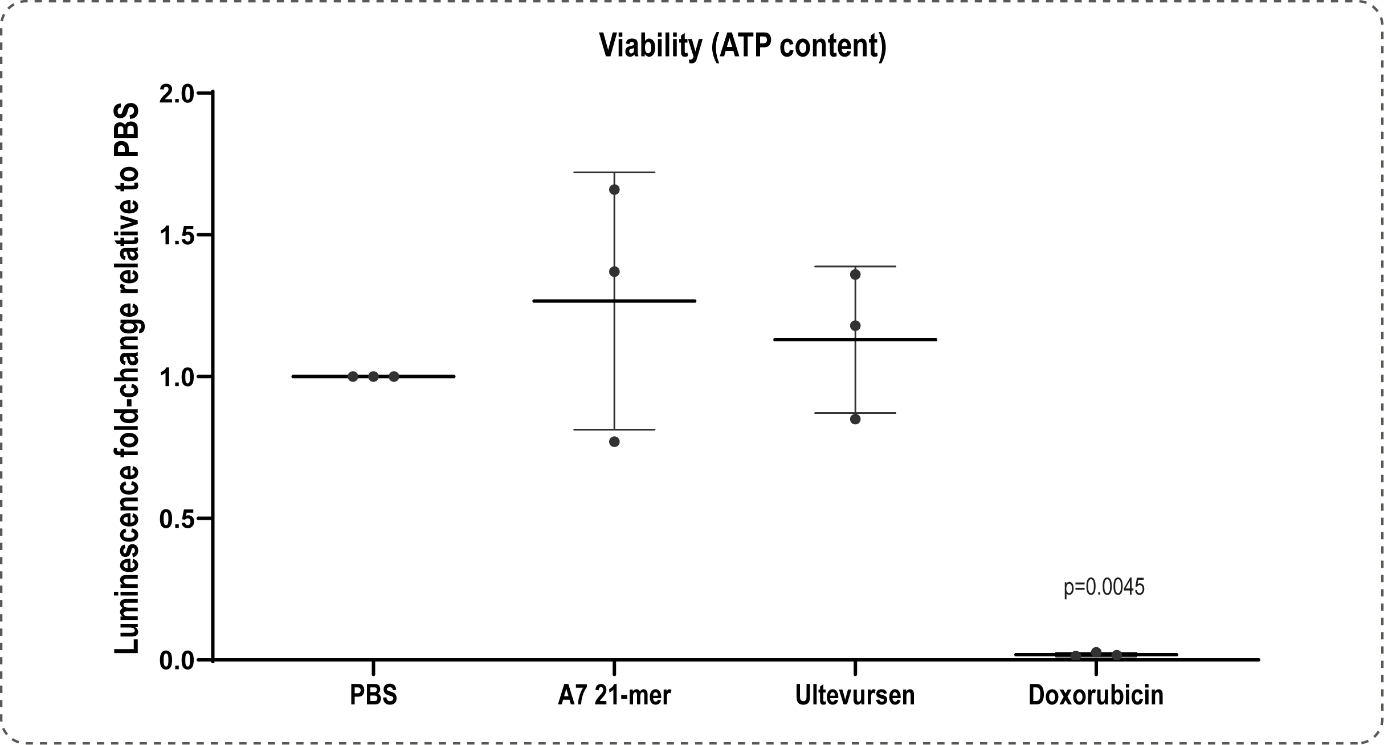


*Supplementary Figure 16* *Toxicity evaluation of A7 21-mer in ROs.*

Viability measured as ATP-content at 10 d post-delivery. Three independent experiments including 3 ROs per experimental condition are presented. DOX : doxorubicin, cytotoxic compound used as positive control. Significance was calculated by one-way ANOVA followed by post-hoc Dunnett test comparing each AON-treated sample to vehicle (PBS).


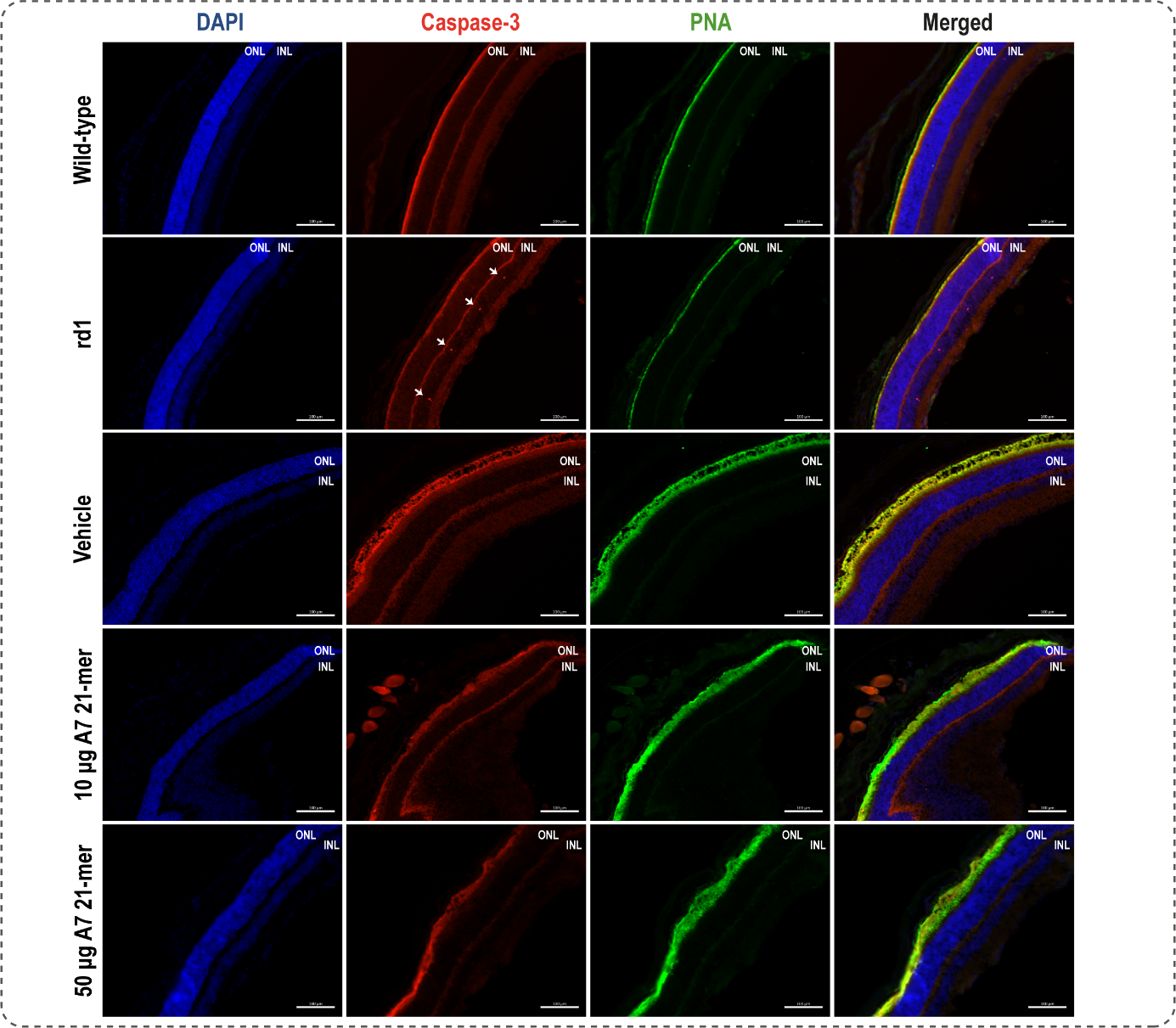


*Supplementary Figure 17 Toxicity evaluation of A7 21-mer in vivo.*

Immunohistochemistry detection of Caspase-3 positive cells (red), counterstained with DAPI (blue) and PNA (outer segments, green). Treated retinas were collected 1 week post-injection. Retina from *rd1* mouse (p11) and its wild-type counterpart (kind gifts from Prof. François Paquet-Durand) was used as positive and negative control, respectively. Representative images from three animals are shown. Scale bar equals 50 μm.


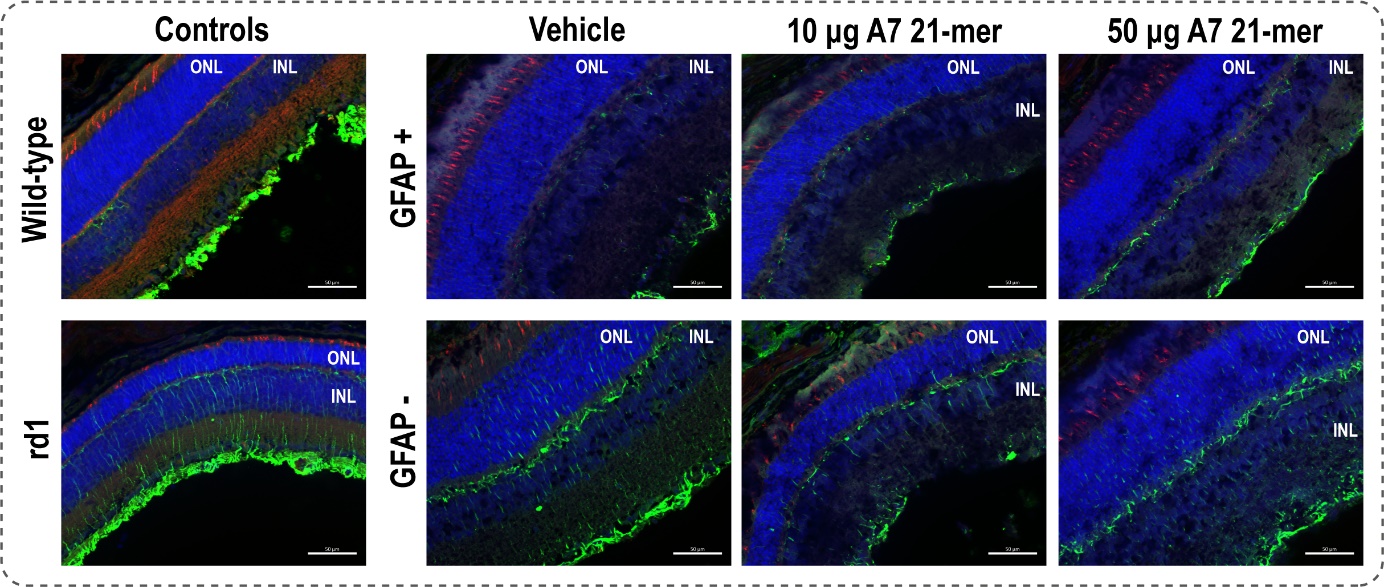


*Supplementary Figure 18 Adverse effect evaluation of A7 21-mer in vivo.* Immunohistochemistry detection of GFAP (green), counterstained with DAPI (blue) and PNA (outer segments, red). Treated retinas were collected 1 week post-injection. Retina from *rd1* mouse (p13) and its wild-type counterpart (kind gifts from Prof. Dr. François Paquet-Durand) was used as positive and negative control, respectively. Representative images from three animals are shown. Scale bar equals 50 μm.

*Supplementary Table 1 Cell culture media composition.*

| **Medium name** | **Basal medium** | **Supplements** |
| --- | --- | --- |
| Fibroblast culture medium | DMEM (Sigma Aldrich) | 20% FCS (Sigma Aldrich, 1% sodium pyruvate (Sigma Aldrich), 1% Pen/Strep (Sigma Aldrich) |
| iPSC culture medium | E8 flex basal medium (Gibco) | E8 flex supplements (Gibco), 1:500 Primocin (Invivogen) |
| HEK293T culture medium | DMEM (Sigma Aldrich) | 10% FCS (Sigma Aldrich, 1% sodium pyruvate (Sigma Aldrich), 1% Pen/Strep (Sigma Aldrich) |
| hTERT RPE-1 culture medium | 1:1 DMEM (Sigma Aldrich) : F12 (Sigma Aldrich) | 10% FCS (Sigma Aldrich, 1% sodium pyruvate (Sigma Aldrich), 1% Pen/Strep (Sigma Aldrich) |
| Neural induction medium | Advanced DMEM/F12 (Gibco) | 1% N2 (Gibco), 1x Glutamax (Gibco), and 1x Primocin (Invivogen) |
| Retinal differentiation medium | 3:1 DMEM (Gibco) : F12 (Gibco) | 2% B27 (Gibco), 1x MEM NEAA (Sigma Aldrich) and 1x Primocin (Invivogen) |
| Retinal maturation medium 1 | 3:1 DMEM (Gibco) : F12 (Gibco) | 10% FCS (Sigma Aldrich), 2% B27 (Gibco), 0.1 μM Taurine (Sigma Aldrich), 1x Glutamax (Gibco) and 1x Primocin (Invivogen) |
| Retinal maturation medium 2 | 3:1 DMEM (Gibco) : F12 (Gibco) | 10% FCS (Sigma Aldrich), 2% B27 (Gibco), 1% N2 (Gibco), 0.1 μM Taurine (Sigma Aldrich), 1x Glutamax (Gibco) and 1x Primocin (Invivogen) |
| Retinal maturation medium 3 | 3:1 DMEM (Gibco) : F12 (Gibco) | 10% FCS (Sigma Aldrich), 1% N2 (Gibco), 0.1 μM Taurine (Sigma Aldrich), 1x Glutamax (Gibco) and 1x Primocin (Invivogen) |
| PBMC culture medium | RPMI 1640 Medium Dutch modification (Gibco) | 0.05 mg/ml Gentamycin (Centraform), 1 mM Sodium Pyruvate (Gibco) and 2 mM GlutaMAX™ (Gibco) |

*Supplementary Table 2 Sequences of primers and probes used in this study. The “{}” sign denotes LNA nucleotide.*

| **Primer name** | **Sequences (5’ – 3’)** |
| --- | --- |
| *ABCA4* exon 6 – 7 F | CGCTCATGGAGTCCCGGACC |
| *ABCA4* exon 6 – 7 R | CCTCCCCAAGATCTCAGATTG |
| *ACTB* F | ACTGGGACGACATGGAGAAG |
| *ACTB* R | TCTCAGCTGTGGTGGTGAAG |
| *ABCA4* mutagenesis F | AGAGACGCGGGGCAAAGACGCTTCCCACACTCCTAGACAG |
| *ABCA4* mutagenesis R | CTGTCTAGGAGTGTGGGAAGCGTCTTTGCCCCGCGTCTCT |
| *POU5F1* F | GCAGCAGATCAGCCACATC |
| *POU5F1* R | CCTCTCGTTGTGCATAGTCG |
| *PAX6* F | GCTGCAAAGAAATAGAACATCC |
| *PAX6* R | TTGGCTGCTAGTCTTTCTCG |
| *SIX6* F | CCAGGCAACCGGACTGAC |
| *SIX6* R | TGTGACAGGACCTGCTGCT |
| *CRX* F | CCCCAGTGTGGATCTGATG |
| *CRX* R | CAAACAGTGCCTCCAGCTC |
| *RCVRN* F | ACACCAAGTTCTCGGAGGAG |
| *RCVRN* R | ACTTGGCGTAGATGCTCTGG |
| *NRL* F | GGCTCCACACCTTACAGCTC |
| *NRL* R | AGCCAGTACAGCTCCTCCAG |
| *OPN1SW* F | TTCTTCTCCAAGAGTGCTTGC |
| *OPN1SW* R | CCTTCCCACACACCATCTTC |
| *RPE65* F | TTACTACGCTTGCACAGAGACC |
| *RPE65* R | GCCCCATTGACAGAGACATAG |
| *ABCA4* F (qPCR) | CATCCTGTTCCACCACCTCA |
| *ABCA4* R (qPCR) | CTGTGTCCTCCAACATGGCT |
| *GUSB* F | AGAGTGGTGCTGAGGATTGG |
| *GUSB* R | CCCTCATGCTCTAGCGTGTC |
| Capture probe | phosphate-AAT{TG}CA{AGG}-TEG-Biotin |
| Detection probe | Digoxigenin-GGCTGCTCGCC |

*Supplementary Table 3 Details of antibodies and ELISA kits used in this study.*

| **Target** | **Antibody** | **Application** |
| --- | --- | --- |
| ABCA4 | Polyclonal rabbit anti-ABCA4 (Abcam ab72955) | WB 1:1000 |
| ABCA4 | Monoclonal mouse anti-ABCA4 clone 3F4 (Abcam ab77285) | IHC 1:100, ICC 1:800 |
| ABCA4 | Monolconal mouse anti-ABCA4 clone 5B4 (Merck MABN2440) | IHC 1:100, ICC 1:400 |
| β-TUBULIN | Polyclonal rabbit anti-β-TUBULIN (Abcam ab15568) | WB 1:1000 |
| HA | Monoclonal mouse anti-HA (Sigma H9658) | WB 1:1000 |
| RHO | Monoclonal mouse anti-RHO clone 4D2 (Novus Biologicals NBP2-59690) | IHC 1:500 |
| ARR3 | Polyclonal goat anti-ARR3 (Novus Biologicals NBP1-37003) | IHC 1:100 |
| CRX | Monoclonal mouse anti-CRX clone 4G11 (Abnova H00001406-M02) | IHC 1:500 |
| GFAP | Polyclonal rabbit anti-GFAP (DAKO Z0334) | IHC 1:1000 |
| Caspase-3 | Polyclonal rabbit anti-active Caspase-3 (BD Pharmingen 559565) | IHC 1:500 |
| Secondary | Goat anti-mouse Alexa Fluor 568 (Thermo Fisher Scientific A11031) | IHC 1:1000, ICC 1:500 |
| Secondary | Goat anti-mouse Alexa Fluor 488 (Thermo Fisher Scientific A11029) | IHC 1:1000, ICC 1:500 |
| Secondary | Goat anti-rabbit Alexa Fluor 568 (Thermo Fisher Scientific A11011) | IHC 1:1000 |
| Secondary | Goat anti-rabbit Alexa Fluor 488 (Thermo Fisher Scientific A11008) | IHC 1:1000 |
| Secondary | Goat anti-rabbit IRDye 800 (Li-Cor LI 926-32211) | WB 1:10,000 |
| Secondary | Goat anti-rabbit Alexa Fluor 680 (Molecular Probes A-21076) | WB 1:10,000 |
| Outer segment | Lectin PNA Alexa Fluor 488 Conjugate (Thermo Fisher Scientific L21409) | IHC 1:100 |
| Outer segment | Lectin PNA Alexa Fluor 568 Conjugate (Thermo Fisher Scientific L32458) | IHC 1:50 |
| Nucleus | DAPI (Invitrogen D1306) | IHC 1:8000 |
| IL-6 | Duoset ELISA Human IL-6 (R&D Systems DY206-5) | According to manual, sample diluted 20x |
| TNFα | Duoset ELISA Human TNFα (R&D Systems DY210-5) | According to manual, sample diluted 5x |
| IFNα | ELISA Flex Human IFNα (MABTECH 3425-1H-6) | According to manual, sample diluted 2x |
| IP-10 | Duoset ELISA Human IP-10 (R&D Systems DY266-5) | According to manual, sample diluted 5x |
| MIP-1α | Duoset ELISA Human MIP-1α (R&D Systems DY270-5) | According to manual, sample diluted 50x |
| MIP-1β | Duoset ELISA Human MIP-1β (R&D Systems DY271-5) | According to manual, sample diluted 50x |

*Supplementary Table 4*  *Sequences of AONs used in this study. The “<>” sign denotes the 2’-O-methoxyethyl chemistry, while the “*” sign represents the phosphorothioate linkage.*

| **AON** | **Sequences (5’ – 3’)** |
| --- | --- |
| A1 | <C*G*A*G*C*A*G*C*C*A*A*A*C*C*C*C*U*C*C*C*U> |
| A2 | <G*G*C*G*A*G*C*A*G*C*C*A*A*A*C*C*C*C*U*C*C> |
| A3 | <U*U*G*G*C*G*A*G*C*A*G*C*C*A*A*A*C*C*C*C*U> |
| A4 | <A*A*U*U*G*G*C*G*A*G*C*A*G*C*C*A*A*A*C*C*C> |
| A5 | <G*C*A*A*U*U*G*G*C*G*A*G*C*A*G*C*C*A*A*A*C> |
| A6 | <U*U*G*C*A*A*U*U*G*G*C*G*A*G*C*A*G*C*C*A*A> |
| A7 (A7 21-mer) | <C*C*U*U*G*C*A*A*U*U*G*G*C*G*A*G*C*A*G*C*C> |
| A8 | <C*A*C*C*U*U*G*C*A*A*U*U*G*G*C*G*A*G*C*A*G> |
| A9 | <U*C*A*C*C*U*U*G*C*A*A*U*U*G*G*C*G*A*G*C*A> |
| A10 | <A*U*C*A*C*C*U*U*G*C*A*A*U*U*G*G*C*G*A*G*C> |
| A11 | <A*A*U*C*A*C*C*U*U*G*C*A*A*U*U*G*G*C*G*A*G> |
| A12 | <G*A*A*U*C*A*C*C*U*U*G*C*A*A*U*U*G*G*C*G*A> |
| A13 | <G*G*A*A*U*C*A*C*C*U*U*G*C*A*A*U*U*G*G*C*G> |
| A14 | <A*G*G*A*A*U*C*A*C*C*U*U*G*C*A*A*U*U*G*G*C> |
| A15 | <C*A*G*G*A*A*U*C*A*C*C*U*U*G*C*A*A*U*U*G*G> |
| A16 | <C*C*A*G*G*A*A*U*C*A*C*C*U*U*G*C*A*A*U*U*G> |
| A17 | <C*C*C*A*G*G*A*A*U*C*A*C*C*U*U*G*C*A*A*U*U> |
| A18 | <C*C*C*C*A*G*G*A*A*U*C*A*C*C*U*U*G*C*A*A*U> |
| A19 | <A*C*C*C*C*A*G*G*A*A*U*C*A*C*C*U*U*G*C*A*A> |
| A20 | <U*A*C*C*C*C*A*G*G*A*A*U*C*A*C*C*U*U*G*C*A> |
| A21 | <C*U*A*C*C*C*C*A*G*G*A*A*U*C*A*C*C*U*U*G*C> |
| A22 | <G*C*U*A*C*C*C*C*A*G*G*A*A*U*C*A*C*C*U*U*G> |
| A23 | <U*G*C*U*A*C*C*C*C*A*G*G*A*A*U*C*A*C*C*U*U> |
| A24 | <C*U*G*C*U*A*C*C*C*C*A*G*G*A*A*U*C*A*C*C*U> |
| A25 | <U*C*U*G*C*U*A*C*C*C*C*A*G*G*A*A*U*C*A*C*C> |
| A7 19-mer | <C*C*U*U*G*C*A*A*U*U*G*G*C*G*A*G*C*A*G> |
| A7 20-mer | <C*C*U*U*G*C*A*A*U*U*G*G*C*G*A*G*C*A*G*C> |
| Scrambled SON | <C*C*U*C*U*U*A*C*C*U*C*A*G*U*U*A*C*A*A*U> |
| SON of A7 21-mer | <G*G*C*T*G*C*T*C*G*C*C*A*A*T*T*G*C*A*A*G*G> |

*Supplementary Data 1 Differential gene expression observed in the following pairwise comparisons, ISO+AON vs ISO+SON, PAT+AON vs PAT+SON, ISO+AON vs PAT AON, and ISO+SON vs PAT+SON. The list is provided as raw data without filtering for any parameter, including adjusted p-value (padj).* *ISO: isogenic control, PAT: patient-derived ROs, AON: A7 21-mer, SON: sense oligonucleotides of A7 21-mer.*

*Supplementary Data 2 Gene ontology enrichment analysis of genes that are differentially expressed in both ISO+AON vs PAT+AON and ISO+SON vs PAT+SON, uniquely in ISO+AON vs PAT+AON and uniquely in ISO+SON vs PAT+SON. ISO: isogenic control, PAT: patient-derived ROs, AON: A7 21-mer, SON: sense oligonucleotides of A7 21-mer.*

*Supplementary Data 3 Differential splicing analysis in the following pairwise comparisons, ISO+AON vs ISO+SON, PAT+AON vs PAT+SON, ISO+AON vs PAT AON, and ISO+SON vs PAT+SON. The list is provided as raw data without filtering for any parameter, including adjusted p-value (padj).* *ISO: isogenic control, PAT: patient-derived ROs, AON: A7 21-mer, SON: sense oligonucleotides of A7 21-mer.*

*Supplementary Data 4 Potential off-targets determined by GGGenome. Allowing up to 3 insertions/deletions.*

*Supplementary Data 5 Potential off-targets determined by GGGenome. Allowing up to 3 mismatches.*
