# Supplementary material for: Preclinical assessment of splicing modulation therapy for ABCA4 variant c.768G>T in Stargardt disease
Source: Commun Med (Lond). 2025 Jan 21;5:25. doi: 10.1038/s43856-024-00712-7 (PMC11751084; doi:10.1038/s43856-024-00712-7)
Supplement: Supplementary file 3 — Description of Additional Supplementary Files [file 43856_2024_712_MOESM3_ESM.pdf]

Description of Additional Supplementary Files

File name: Supplementary Data 1

File description: Differential gene expression observed in the following pairwise comparisons, ISO+AON vs ISO+SON, PAT+AON vs PAT+SON, ISO+AON vs PAT AON, and ISO+SON vs PAT+SON. The list is provided as raw data without filtering for any parameter, including adjusted p-value (padj). ISO: isogenic control, PAT: patient-derived ROs, AON: A7 21-mer, SON: sense oligonucleotides of A7 21-mer.

File name: Supplementary Data 2

File description: Gene ontology enrichment analysis of genes that are differentially expressed in both ISO+AON vs PAT+AON and ISO+SON vs PAT+SON, uniquely in ISO+AON vs PAT+AON and uniquely in ISO+SON vs PAT+SON. ISO: isogenic control, PAT: patient derived ROs, AON: A7 21-mer, SON: sense oligonucleotides of A7 21-mer.

File name: Supplementary Data 3

File description: Differential splicing analysis in the following pairwise comparisons, ISO+AON vs ISO+SON, PAT+AON vs PAT+SON, ISO+AON vs PAT AON, and ISO+SON vs PAT+SON. The list is provided as raw data without filtering for any parameter, including adjusted p-value (padj). ISO: isogenic control, PAT: patient-derived ROs, AON: A7 21-mer, SON: sense oligonucleotides of A7 21-mer.

File name: Supplementary Data 4

File description: Potential off-targets determined by GGGenome. Allowing up to 3 insertions/deletions.

File name: Supplementary Data 5

File description: Potential off-targets determined by GGGenome. Allowing up to 3 mismatches.
