# Supplementary material for: Preclinical assessment of splicing modulation therapy for ABCA4 variant c.768G>T in Stargardt disease
Source: Commun Med (Lond). 2025 Jan 21;5:25. doi: 10.1038/s43856-024-00712-7 (PMC11751084; doi:10.1038/s43856-024-00712-7)
Supplement: Supplementary file 10 — Reporting Summary [file 43856_2024_712_MOESM10_ESM.pdf]

nature portfolio

Corresponding author(s): Rob W.J. Collin

Last updated by author(s): Nov 25, 2024

Reporting Summary

Nature Portfolio wishes to improve the reproducibility of the work that we publish. This form provides structure for consistency and transparency in reporting. For further information on Nature Portfolio policies, see our Editorial Policies and the Editorial Policy Checklist.

Statistics

For all statistical analyses, confirm that the following items are present in the figure legend, table legend, main text, or Methods section.

n/a	Confirmed	
☐	☒	The exact sample size (*n*) for each experimental group/condition, given as a discrete number and unit of measurement
☐	☒	A statement on whether measurements were taken from distinct samples or whether the same sample was measured repeatedly
☐	☒	The statistical test(s) used AND whether they are one- or two-sided<br>*Only common tests should be described solely by name; describe more complex techniques in the Methods section.*
☒	☐	A description of all covariates tested
☐	☒	A description of any assumptions or corrections, such as tests of normality and adjustment for multiple comparisons
☐	☒	A full description of the statistical parameters including central tendency (e.g. means) or other basic estimates (e.g. regression coefficient) AND variation (e.g. standard deviation) or associated estimates of uncertainty (e.g. confidence intervals)
☒	☐	For null hypothesis testing, the test statistic (e.g. *F*, *t*, *r*) with confidence intervals, effect sizes, degrees of freedom and *P* value noted<br>*Give P values as exact values whenever suitable.*
☒	☐	For Bayesian analysis, information on the choice of priors and Markov chain Monte Carlo settings
☒	☐	For hierarchical and complex designs, identification of the appropriate level for tests and full reporting of outcomes
☒	☐	Estimates of effect sizes (e.g. Cohen's *d*, Pearson's *r*), indicating how they were calculated

Our web collection on statistics for biologists contains articles on many of the points above.

Software and code

Policy information about availability of computer code

Data collection	n.a.
Data analysis	n.a.

For manuscripts utilizing custom algorithms or software that are central to the research but not yet described in published literature, software must be made available to editors and reviewers. We strongly encourage code deposition in a community repository (e.g. GitHub). See the Nature Portfolio guidelines for submitting code & software for further information.

Data

Policy information about availability of data

All manuscripts must include a data availability statement. This statement should provide the following information, where applicable:

- Accession codes, unique identifiers, or web links for publicly available datasets
- A description of any restrictions on data availability
- For clinical datasets or third party data, please ensure that the statement adheres to our policy

The source data of Figure 2c, 2e, 3c, 4c, 6a and 6b are available in Supplementary Data 6. For Figure 5, the source data are available in Supplementary Data 1 and 3. The raw RNA-seq data were deposited in Gene Expression Omnibus with accession number GSE253344. ThOthere datasets used and/or analysed during the current study are available from the corresponding author on reasonable request.

Human research participants

Policy information about studies involving human research participants and Sex and Gender in Research.

Reporting on sex and gender	n.a.
Population characteristics	n.a.
Recruitment	n.a.
Ethics oversight	n.a.

Note that full information on the approval of the study protocol must also be provided in the manuscript.

Field-specific reporting

Please select the one below that is the best fit for your research. If you are not sure, read the appropriate sections before making your selection.

☒ Life sciences ☐ Behavioural & social sciences ☐ Ecological, evolutionary & environmental sciences

For a reference copy of the document with all sections, see nature.com/documents/nr-reporting-summary-flat.pdf

Life sciences study design

All studies must disclose on these points even when the disclosure is negative.

Sample size	Sample size varies depending on the experimental requirements. These are indicated in each figure legends.
Data exclusions	No data was excluded from the analysis
Replication	Each in vitro result shown in this study is reproduced at least once. In vivo work was performed once as a pilot.
Randomization	Retinal organoids and mice were randomly allocated into the experimental groups.
Blinding	Blinding was applied to the in vivo experiments. No blinding was applied to the in vitro work.

Reporting for specific materials, systems and methods

We require information from authors about some types of materials, experimental systems and methods used in many studies. Here, indicate whether each material, system or method listed is relevant to your study. If you are not sure if a list item applies to your research, read the appropriate section before selecting a response.

Materials & experimental systems

n/a	Involved in the study	
☐	☒	Antibodies
☐	☒	Eukaryotic cell lines
☒	☐	Palaeontology and archaeology
☐	☒	Animals and other organisms
☒	☐	Clinical data
☒	☐	Dual use research of concern

Methods

n/a	Involved in the study	
☐	☐	ChIP-seq
☐	☐	Flow cytometry
☐	☐	MRI-based neuroimaging

Antibodies

Antibodies used	These are indicated in Table S3
Validation	Each antibody was thoroughly tested with appropriate negative controls per application.

Eukaryotic cell lines

Policy information about cell lines and Sex and Gender in Research

Cell line source(s)	Induced pluripotent stem cells were patient-derived, whereas HEK293T and hTERT-RPE1 were from ATCC.
Authentication	Induced pluripotent stem cells authentication can be seen in ref 8 and 9
Mycoplasma contamination	The cells used in this study were routinely tested for mycoplasma and showed negative results.
Commonly misidentified lines (See ICLAC register)	*Name any commonly misidentified cell lines used in the study and provide a rationale for their use.*

Animals and other research organisms

Policy information about studies involving animals; ARRIVE guidelines recommended for reporting animal research, and Sex and Gender in Research

Laboratory animals	C57BL/6J mice
Wild animals	n.a.
Reporting on sex	n.a.
Field-collected samples	n.a.
Ethics oversight	The mouse experiments were approved by the local Animal Experimentation Committee (RU-DEC-2016-0050, work protocol 2016-0050-022 and 2016-0050-024) and were performed according to the Dutch law (Wet op Dierproeven 1996).

Note that full information on the approval of the study protocol must also be provided in the manuscript.

ChIP-seq

Data deposition

- [ ] Confirm that both raw and final processed data have been deposited in a public database such as GEO.
- [ ] Confirm that you have deposited or provided access to graph files (e.g. BED files) for the called peaks.

Data access links *May remain private before publication.*	n.a.
Files in database submission	n.a.
Genome browser session (e.g. UCSC)	n.a.

Methodology

Replicates	n.a.
Sequencing depth	n.a.
Antibodies	n.a.
Peak calling parameters	n.a.
Data quality	n.a.
Software	n.a.

Flow Cytometry

Plots

Confirm that:

- [ ] The axis labels state the marker and fluorochrome used (e.g. CD4-FITC).
- [ ] The axis scales are clearly visible. Include numbers along axes only for bottom left plot of group (a 'group' is an analysis of identical markers).
- [ ] All plots are contour plots with outliers or pseudocolor plots.
- [ ] A numerical value for number of cells or percentage (with statistics) is provided.

Methodology

Sample preparation	n.a.
Instrument	n.a.
Software	n.a.
Cell population abundance	n.a.
Gating strategy	n.a.

- [ ] Tick this box to confirm that a figure exemplifying the gating strategy is provided in the Supplementary Information.

Magnetic resonance imaging

Experimental design

Design type	n.a.
Design specifications	n.a.
Behavioral performance measures	n.a.

Acquisition

Imaging type(s)	n.a.
Field strength	n.a.
Sequence & imaging parameters	n.a.
Area of acquisition	n.a.

Diffusion MRI ☐ Used ☐ Not used

Preprocessing

Preprocessing software	n.a.
Normalization	n.a.
Normalization template	n.a.
Noise and artifact removal	n.a.
Volume censoring	n.a.

Statistical modeling & inference

Model type and settings	n.a.
Effect(s) tested	n.a.

Specify type of analysis: ☐ Whole brain ☐ ROI-based ☐ Both

Statistic type for inference (See Eklund et al. 2016)	n.a.
Correction	n.a.

Models & analysis

n/a	Involved in the study
☒	☐ Functional and/or effective connectivity
☒	☐ Graph analysis
☒	☐ Multivariate modeling or predictive analysis
